# Influence of Body Mass Index on the Prognostic Value of Tumor ¹⁸F-FDG Uptake in Stage I Non-Small Cell Lung Cancer

**DOI:** 10.1371/journal.pone.0145020

**Published:** 2015-12-18

**Authors:** Seung Hyup Hyun, Kyung-Han Lee, Joon Young Choi, Byung-Tae Kim, Jhingook Kim, Jae Ill Zo, Hojoong Kim, O. Jung Kwon, Hee Kyung Ahn

**Affiliations:** 1 Department of Nuclear Medicine, Samsung Medical Center, Sungkyunkwan University School of Medicine, Seoul, Republic of Korea; 2 Department of Thoracic Surgery, Samsung Medical Center, Sungkyunkwan University School of Medicine, Seoul, Republic of Korea; 3 Division of Pulmonary and Critical Care Medicine, Department of Medicine, Samsung Medical Center, Sungkyunkwan University School of Medicine, Seoul, Republic of Korea; 4 Division of Hematology-Oncology, Department of Internal Medicine, Gachon University Gil Medical Center, Incheon, Republic of Korea; INRS, CANADA

## Abstract

**Background:**

The impact of host energy balance status on outcome of lung cancer has not been fully explored. It is also unknown if there is a potential modifying effect of body mass index (BMI) on tumor cell behavior in patients with early-stage non-small cell lung cancer (NSCLC). We therefore investigated the interactive effects of tumor [^18^F]-fluorodeoxyglucose (FDG) avidity and BMI.

**Methods:**

We investigated 1,197 patients with stage I NSCLC who underwent preoperative FDG positron emission tomography followed by curative resection. The primary outcome measure was disease-free survival (DFS). A multivariable Cox proportional hazards model was used to assess the potential independent effects of the prognostic variables. A stratified Cox regression analysis was also performed to assess the potential modifying effects of BMI on the relationship between tumor FDG uptake and patient survival.

**Results:**

There were 145 tumor recurrences and 19 deaths during a median follow-up of 30 months. Tumor-related variables, including tumor size, maximum standardized uptake value (SUVmax), histologic cell type, differentiation, lymphovascular invasion, and visceral pleural invasion, did not differ significantly according to BMI status. In multivariable Cox regression analysis, overweight or obesity [hazard ratio (HR), 0.59; 95% CI, 0.43–0.81; *P* = 0.001] and tumor SUVmax (HR, 1.72; 95% CI, 1.43–2.07; *P* < 0.001) were significantly associated with DFS. There was a significant modifying effect of BMI (*P* for interaction < 0.001 in multivariable analysis). High tumor SUVmax was more strongly associated with worse DFS in normal weight patients (HR, 4.72; 95% CI, 2.77–8.06; *P* < 0.001) than in overweight or obese patients (HR, 2.61; 95% CI, 1.58–4.31; *P* < 0.001).

**Conclusions:**

Tumor FDG avidity is an independent predictor of DFS in patients with early-stage NSCLC and this prognostic value was strengthened in normal weight patients than in overweight or obese patients. These results suggest that the host-tumor interaction between host energy balance status and tumor glucose metabolism plays an important role in the outcome of early-stage NSCLC.

## Introduction

Overweight and obesity are serious public health problems with an increasing global prevalence. It is becoming increasingly apparent that these statuses increase the risks of various cancers, including those of the pancreas, colon, breast, endometrium, and esophagus [1], and they are often associated with worse prognoses when cancer occurs. However, overweight or obesity does not adversely affect all types of cancers. In contrast to most other types of malignancies, the incidence of non-small cell lung cancer (NSCLC) is lower in obese individuals [2, 3]. Furthermore, obese patients with NSCLC are reported to have better survival outcomes compared to NSCLC patients of normal weight [4–7]. This counterintuitive phenomenon, referred to as the obesity paradox, supports the hypothesis that excess energy balance can be protective and associated with improved survival for certain diseases. With regard to this phenomenon, there is recent evidence suggesting that the tumors of obese patients may actually possess less aggressive biological characteristics. In a recent study by Hakimi and coworkers, renal cell carcinomas in obese patients were found to have characteristic gene expression profiles of tumor metabolism that may confer a survival advantage to the patients [8]. The association of obesity with better prognosis in renal cell carcinoma was also shown in a recent meta-analysis [9].

[^18^F]-Fluorodeoxyglucose (FDG) positron emission tomography/computed tomography (PET/CT) allows the noninvasive visualization of the metabolic characteristics of tumors in living subjects. Tumor FDG uptake represents the heightened glycolytic metabolism of cancer cells and is widely exploited for cancer detection and staging using PET/CT. In NSCLC, high tumor FDG uptake is associated with greater tumor aggressiveness [10, 11] and poor patient outcome [12, 13]. Given the potential relationship of host energy balance with tumor cell metabolism [14, 15], it is of significant clinical relevance to assess the tumor cell behavior differs between patients of normal weight and those who are overweight or obese.

Despite the relative wealth of data on the beneficial effects of overweight/obesity and adverse effects of tumor FDG avidity in patients with NSCLC, none of the previous studies examined a potential modifying effect of host energy balance status on tumor cell behavior in patients with early-stage NSCLC. We therefore investigated the interactive effects of tumor FDG avidity and overweight/obesity, measured as body mass index (BMI) in patients with early-stage NSCLC following curative resection.

## Patients and Methods

### Study Population

This study was approved by the Samsung Medical Center Institutional Review Board and written informed consent was waived. Patient information was anonymized and de-identified prior to analysis. The study cohort consisted of all patients with newly diagnosed, pathologically confirmed early stage NSCLC (stage I, TNM-7 lung cancer staging system), who underwent preoperative FDG PET/CT at our institution from 2008 to 2012. Patients were required to have undergone tumor resection with curative intent and to have a negative resection margin. Patients were excluded if they had received neoadjuvant or adjuvant therapy. A total of 1,197 patients met all inclusion criteria and comprised the study cohort. All subjects were clinically and radiologically followed up according to our institution’s protocol. All patients were evaluated with chest CT or FDG PET/CT performed on an alternating basis every six months for two years after surgery. Thereafter, patients made annual visits to the clinic for surveillance, and FDG PET/CT imaging was performed if clinically indicated.

Demographic and clinical characteristics as well as survival data were obtained from medical records and the institutional tumor registry. Tumor histology, pathological tumor size, and tumor invasion status were obtained from surgical pathology reports. BMI was defined as weight divided by the square of height measured at the time of PET/CT imaging. According to the criteria for Asian populations, the definitions of normal weight, overweight and obesity are BMI < 23.0, 23.0–24.9, and ≥ 25.0 kg/m^2^, respectively [16]. In this study, patients were stratified into two BMI groups, overweight/obesity (high BMI, ≥ 23.0 kg/m^2^) and normal weight (low BMI, < 23.0 kg/m^2^).

### FDG PET/CT Imaging

Patients fasted for at least 6 h before the PET/CT study. Blood glucose level was measured and was required to be less than 200 mg/dL. Whole-body PET and unenhanced CT images were acquired with arms down for patient comfort using a PET/CT scanner (Discovery STE, GE Healthcare, Waukesha, WI, USA) 60 min after injecting FDG (5.0 MBq/kg). CT was performed using a 16-slice helical CT scanner with 30–170 mAs adjusted to patient body weight at 140 kVp with a 3.75 mm section width. This scan was followed by an emission scan from the thigh to the head at 2.5 min/frame in three-dimensional mode. PET images were reconstructed using CT for attenuation correction with an ordered subset expectation maximization algorithm (20 subsets and 2 iterations) and a voxel size of 3.9 × 3.9 × 3.3 mm. For semi-quantitative analysis, maximum standardized uptake value (SUV) of FDG uptake was measured by manually placing a spherical volume of interest over the primary tumor. Two different normalization methods were used to measure tumor FDG uptake. SUVmax normalized to patient body weight (SUVbw) was used as a more popular index, while SUV normalized to body surface area (SUVbsa) was used as an index that can account for different patient size.

### Statistical Analyses

Our primary outcome measure was disease-free survival (DFS), defined as the time between the date of surgery and the first local or distant recurrence of the index lung cancer. Variables for the survival analyses included age at diagnosis, sex, smoking status (never-smoker vs. ever-smoker), histological cell type (non-squamous cell carcinoma vs. squamous cell carcinoma), tumor differentiation (well or moderately differentiated vs. poorly differentiated), pathological tumor size, lymphovascular invasion, visceral pleural invasion, type of resection (lobectomy or pneumonectomy vs. limited resection less than lobectomy), BMI (overweight or obesity vs. normal weight), and primary tumor SUV.

Univariable Cox proportional hazards models were used to determine the hazard ratios (HRs) for selected potential prognostic factors. A multivariable Cox proportional hazards model was used to assess the potential independent effects of the prognostic variables after adjusting for known risk factors. A stratified Cox regression analysis was also performed to assess the potential modifying effects of BMI. An interaction was assessed by including the cross product of BMI and tumor SUV in a multivariable Cox model, and the Wald test was performed. Associations of clinical features with BMI were tested using Chi-square test (categorical variables) or two-sample *t*-test (continuous variables). Survival curves were estimated using the Kaplan–Meier method, and the differences between subgroups were compared using the log-rank test. Patients were stratified as high and low tumor SUV groups using an optimal cutoff based on maximally selected rank statistics [17]. All tests were two-sided, and *P*-values less than 0.05 were considered significant.

## Results

**Fig 1:**
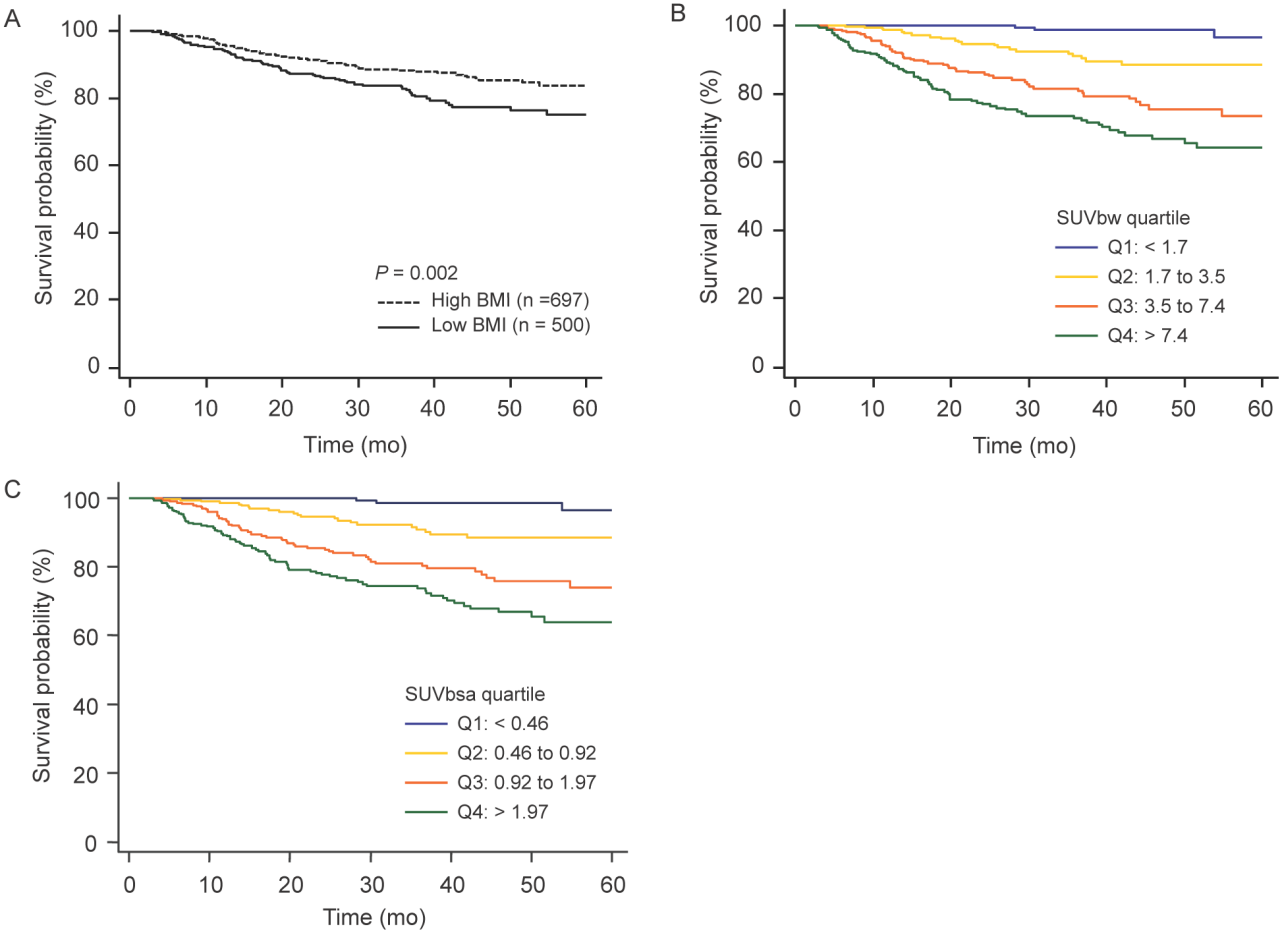
Disease-free survival curves of 1,197 patients with stage I NSCLC according to (A) BMI, (B) tumor SUVbw, and (C) tumor SUVbsa. NSCLC, non-small cell lung cancer; BMI, body mass index; SUVbw, maximum standardized uptake value normalized to body weight; SUVbsa, maximum standardized uptake value normalized to body surface area.

The characteristics of our cohort of 1,197 patients (708 males and 489 females; mean age, 62 years; range, 16–89 years) are summarized in Table 1. Clinical characteristics and tumor-related variables were also compared with subjects categorized according to BMI. There were no significant differences between BMI groups in age, sex, smoking status, T stage, tumor size, SUVbw, histologic cell type, tumor differentiation, lymphovascular invasion, or visceral pleural invasion. There was a significant difference in tumor SUVbsa between low and high BMI groups (Table 1). Kaplan–Meier analysis of the entire cohort demonstrated significantly improved survival in patients with higher BMI and lower tumor SUV (Fig 1).

**Table 1:** Clinical Characteristics of Study Subjects Categorized by BMI Status.

Characteristic	Entire cohort (n = 1197)	Low BMI (n = 500)	High BMI (n = 697)	*P*
Age (years)	61.8 ± 9.9	61.5 ± 10.8	61.9 ± 9.2	0.456
Sex, male	708 (59.1)	287 (57.4)	421 (60.4)	0.311
BMI (kg/m^2^)	23.6 ± 2.8	21.0 ± 1.5	25.5 ± 2.0	< 0.001
< 18.5	39 (3.3)	39 (7.8)		
18.5–22.9	461 (38.5)	461 (92.2)		
23.0–24.9	352 (29.4)		352 (50.5)	
25.0–29.9	324 (27.1)		324 (46.5)	
≥ 30.0	21 (1.8)		21 (3.0)	
Smoking status				0.241
Never-smoker	607 (50.7)	264 (52.8)	343 (49.2)	
Ever-smoker (current or former)	590 (49.3)	236 (47.2)	354 (50.8)	
Histological cell type				0.339
Non-squamous cell carcinoma	1004 (83.9)	413 (82.6)	591 (84.8)	
Squamous cell carcinoma	193 (16.1)	87 (17.4)	106 (15.2)	
Tumor differentiation				0.915
Well or moderate	1098 (91.7)	458 (91.6)	640 (91.8)	
Poor	99 (8.3)	42 (8.4)	57 (8.2)	
Pathologic T stage				0.108
T1a	536 (44.8)	206 (41.2)	330 (47.3)	
T1b	325 (27.2)	145 (29.0)	180 (25.8)	
T2a	336 (28.0)	149 (29.8)	187 (26.8)	
Pathologic tumor size (cm)	2.3 ± 0.9	2.3 ± 0.9	2.2 ± 0.9	0.349
Lymphovascular invasion	269 (22.5)	110 (22.0)	159 (22.8)	0.779
Visceral pleural invasion	135 (11.3)	47 (9.4)	88 (12.6)	0.095
Type of resection				0.860
Wedge-segmentectomy	152 (12.7)	62 (12.4)	90 (12.9)	
Lobectomy-pneumonectomy	1045 (87.3)	438 (87.6)	607 (87.1)	
Tumor SUVbw	5.2 ± 4.8	5.3 ± 4.8	5.2 ± 4.9	0.666
Tumor SUVbsa	1.4 ± 1.3	1.5 ± 1.4	1.3 ± 1.3	0.013

Data are numbers of patients (percentage) or mean ± standard deviation.

BMI, body mass index; SUVbw, maximum standardized uptake value normalized to body weight; SUVbsa, maximum standardized uptake value normalized to body surface area

During a median follow-up duration of 30 months, a total of 145 tumor recurrences (19.8%) and 19 patient deaths occurred. In univariable survival analysis, age, tumor differentiation, pathological tumor size, lymphovascular invasion, visceral pleural invasion, tumor SUVbw, and overweight/obesity were significant prognostic factors for DFS (Table 2). In multivariable Cox regression analysis after adjusting for all potential prognostic variables, lymphovascular invasion (HR, 2.34; 95% CI, 1.68–3.27; *P* < 0.001), visceral pleural invasion (HR, 2.25; 95% CI, 1.55–3.27; *P* < 0.001), overweight/obesity (HR, 0.59; 95% CI, 0.43–0.81; *P* = 0.001), and tumor SUVbw (HR, 1.72; 95% CI, 1.43–2.07; *P* < 0.001) were significantly associated with DFS (Table 2). Tumor SUVbsa was also a significantly associated with DFS (HR, 1.69; 95% CI, 1.40–2.04; *P* < 0.001; S1 Table).

**Table 2:** Disease-Free Survival in Univariable and Multivariable Analyses of the Entire Cohort (n = 1,197).

	Univariable analysis			Multivariable analysis		
Clinicopathologic Variables	HR	95% CI	*P*	HR	95% CI	*P*
Age (1-y increase)	1.04	1.02–1.06	< 0.001	1.01	0.99–1.03	0.073
Sex, male vs. female	1.69	1.19–2.39	0.003	1.48	0.89–2.46	0.128
Smoking status						
Ever-smoker vs. never-smoker	1.56	1.13–2.16	0.006	0.81	0.51–1.30	0.402
Overweight/obesity *vs*. normal weight	0.61	0.45–0.84	0.003	0.59	0.43–0.81	0.001
Histologic cell type						
Squamous vs. non-squamous	2.05	1.44–2.92	< 0.001	0.85	0.56–1.27	0.434
Tumor differentiation						
Poor *vs*. well or moderate	2.67	1.76–4.05	< 0.001	1.21	0.78–1.87	0.389
Pathological tumor size (cm)	1.68	1.46–1.94	< 0.001	1.09	0.90–1.31	0.366
Lymphovascular invasion	3.89	2.84–5.34	< 0.001	2.34	1.68–3.27	< 0.001
Visceral pleural invasion	2.82	1.97–4.03	< 0.001	2.25	1.55–3.27	< 0.001
Limited resection less than lobectomy	0.47	0.24–0.93	0.032	1.21	0.59–2.49	0.587
Tumor SUVbw (continuous, log_2_ scale)	1.97	1.72–2.27	< 0.001	1.72	1.43–2.07	< 0.001

NSCLC, non-small cell lung cancer; HR, hazard ratio; CI, confidence interval; SUVbw, maximum standardized uptake value normalized to body weight

**Fig 2:**
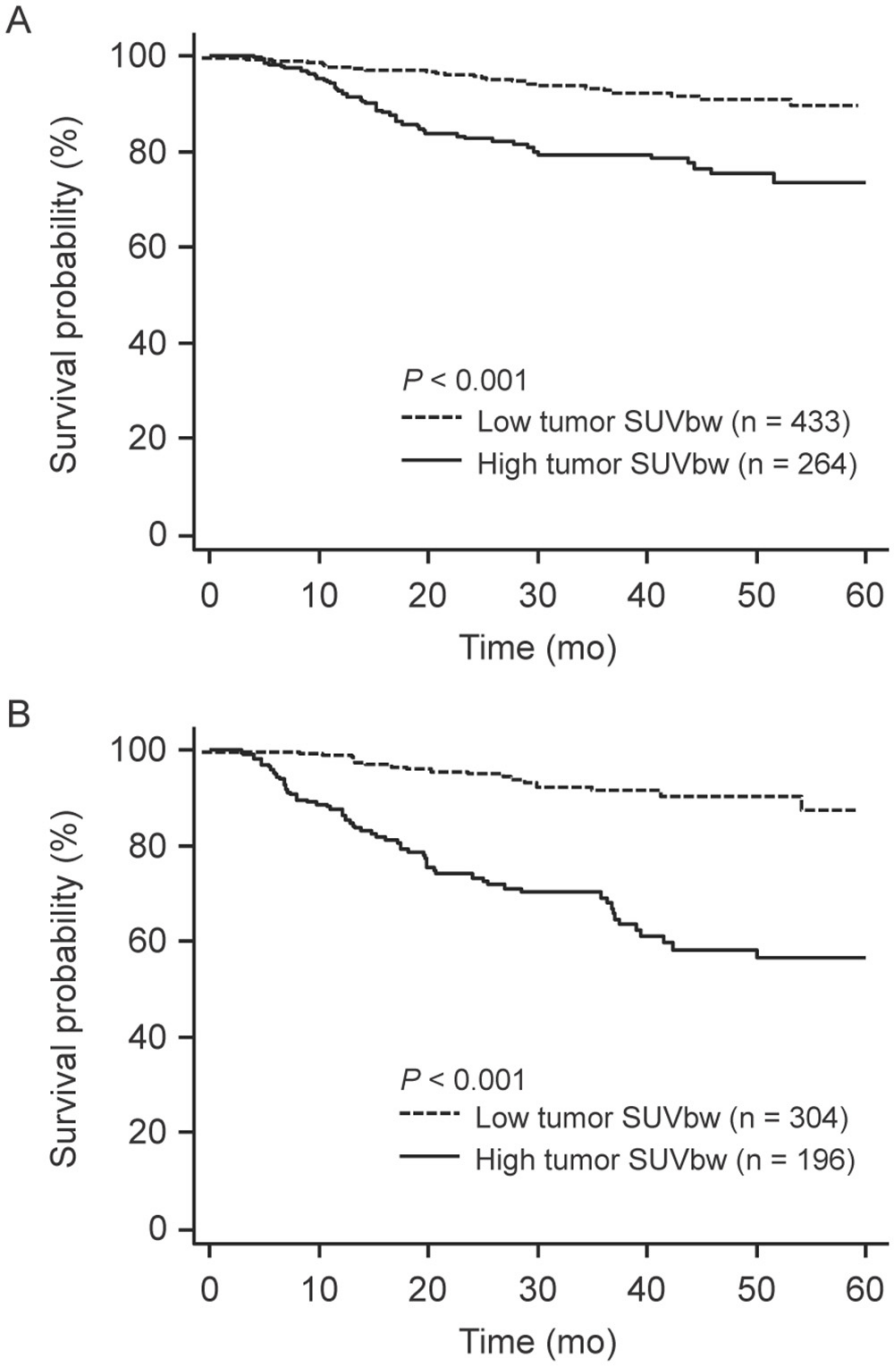
Disease-free survival curves of (A) 697 overweight/obese and (B) 500 normal weight patients with stage I NSCLC according to tumor FDG uptake. NSCLC, non-small cell lung cancer; SUVbw, maximum standardized uptake value normalized to body weight.

**Fig 3:**
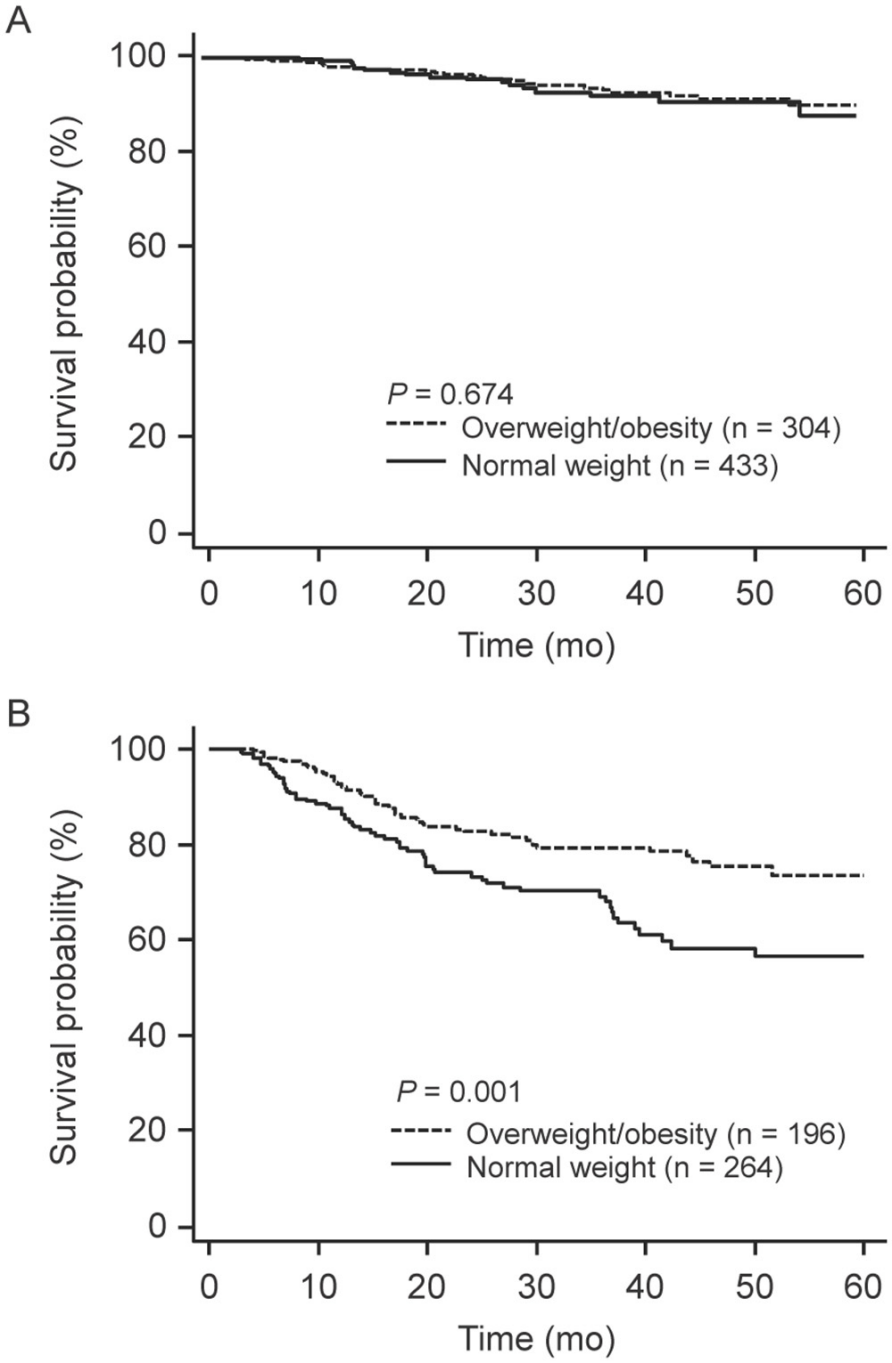
Disease-free survival curves of 1,197 patients with stage I NSCLC according to BMI status in patients with (A) low tumor FDG uptake (SUVbw ≤ 5) and (B) high tumor FDG uptake (SUVbw > 5). NSCLC, non-small cell lung cancer; SUVbw, maximum standardized uptake value normalized to body weight.

We examined the potential modifying effect of BMI on the relationship between tumor SUVbw and patient survival (Table 3). There was a significant modifying effect of BMI (P for interaction < 0.001 in multivariable analysis). High tumor SUVmax (> 5) was more strongly associated with worse DFS in normal weight patients (HR, 4.72; 95% CI, 2.77–8.06; *P* < 0.001) than in overweight or obese patients (HR, 2.61; 95% CI, 1.58–4.31; *P* < 0.001). The differential effects of tumor SUVbw and BMI status on DFS were also observed in Kaplan-Meier analyses (Figs 2 and 3).

**Table 3:** Disease-Free Survival according to Body Mass Index Status and Tumor SUVbw in Stage I NSCLC Patients (n = 1,197).

	Univariable HR (95% CI)	*P*	Multivariable HR* (95% CI)	*P*
Normal weight				
Low tumor SUVbw ≤ 5 (n = 304)	1.00 (referent)		1.00 (referent)	
High tumor SUVbw > 5 (n = 196)	5.93 (3.50–10.01)	< 0.001	4.72 (2.77–8.06)	< 0.001
Overweight/obesity				
Low tumor SUVbw ≤ 5 (n = 433)	1.00 (referent)		1.00 (referent)	
High tumor SUVbw > 5 (n = 264)	3.61 (2.23–5.83)	< 0.001	2.61 (1.58–4.31)	< 0.001
P for interaction		0.002		< 0.001

*The multivariable Cox regression model stratified by body mass index status included lymphovascular invasion, visceral pleural invasion, and tumor SUV variables.

NSCLC, non-small cell lung cancer; SUVbw, maximum standardized uptake value normalized to body weight; HR, hazard ratio; CI, confidence interval

## Discussion

In patients with stage I NSCLC who underwent curative resection, we found that overweight or obesity was independently associated with better DFS, whereas high tumor FDG uptake was independently associated with worse DFS. We found a possible modifying effect of BMI on the relationship between tumor SUV and patient survival. High tumor SUV was more strongly associated with worse DFS in normal weight patients than in overweight or obese patients.

Overweight or obesity is linked to more adverse rather than better outcomes in patients with many different cancers [1, 18–20]. This result has been explained by the increased circulating insulin and insulin-like growth factor (IGF) levels that promote cancer growth in obese subjects [21, 22]. In addition, overweight or obesity results in a higher level of adipocyte-derived leptin, which promotes cell proliferation, and a lower level of adiponectin, which may have anti-proliferative effects. Other possible mechanisms include the effects of fat cells on tumor growth-regulating pathways, low-level inflammation that stimulates tumor growth, and altered immune responses due to obesity [23, 24]. Despite such diverse ways in which overweight or obesity can adversely influence cancer survival, our overweight and obese patients had a significant survival advantage. Several studies have reported a similar inverse relationship between BMI and lung cancer prognosis [4–7]. Our results extend the beneficial effects of overweight or obesity to patients with early-stage NSCLC undergoing curative resection.

In our results, tumor-related variables such as tumor size, tumor SUV, histologic cell type, tumor differentiation, lymphovascular invasion, and visceral pleural invasion were not significantly different according to BMI status. Thus, it is unclear why a normal energy balance status strengthened the link between high tumor glucose metabolism and patient outcome. A possible explanation is a host-tumor interaction between host energy balance status and tumor glucose metabolism, which may modify tumor cell behavior. It is tempting to postulate that malignant cells with a higher glycolytic rate in normal weight patients are more sensitive to tumor promoting signals or are more aggressive than in overweight or obese patients, which might be derived from molecular changes induced by the host energy balance status. However, this speculation requires further investigations for clarification.

Nonetheless, our results have significant clinical implications. First, they demonstrate that tumor FDG uptake is a useful prognostic indicator of early-stage NSCLC, in addition to lymphovascular and visceral pleural invasion status. Secondly, normal weight patients with high tumor FDG uptake have a high risk of recurrence following curative resection and require closer follow-up. Although adjuvant treatment for stage I NSCLC after curative surgery with a negative resection margin is not generally indicated, our findings may help to identify high-risk patients who could benefit from adjuvant chemotherapy following surgery [25, 26]. Finally, a better understanding of how energy balance status and tumor glucose metabolism potentially interact provides insight useful for developing novel metabolism-targeted cancer treatment strategies.

The limitations of this study include its retrospective nature and the lack of tumor biology data such as gene mutation and expression as well as laboratory data regarding serum IGF1, insulin, and adipocyte-derived leptin levels. Our patients were all Asians, who differ in body composition and overweight/obesity criteria from Western populations. According to the criteria for Asian populations, we stratified the patients into two BMI groups, overweight or obesity (BMI ≥ 23.0 kg/m^2^) and normal weight (BMI < 23.0 kg/m^2^) [16]. We combined overweight and obese patients into a single group because there was no survival difference between obese (BMI ≥ 25.0 kg/m^2^) and overweight (BMI 23.0–24.9 kg/m^2^) patients (*P* = 0.805). The number of patients who were underweight (BMI < 18.5 kg/m^2^) or severely obese (BMI ≥ 30 kg/m^2^) was too small for separate analysis of additional categories. Therefore, caution is required when applying our results to other ethnic groups. In addition, simple measurement of FDG uptake using SUV has several limitations compared to more sophisticated quantitative techniques, including susceptibility to influence by various factors. Normalization methods can also affect estimates of FDG uptake. For instance, normalization to body weight can lead to underestimation of FDG uptake in normal weight patients and over-estimation in overweight or obese patients. In our study population, we observed similar prognostic values for SUVbw and SUVbsa. However, it should be noted that normalization to body surface area can be a better choice under certain circumstances [27]

## Conclusions

Tumor FDG avidity is an independent predictor of adverse outcome in patients with early-stage NSCLC, and this prognostic value was strengthened in normal weight patients than in overweight or obese patients. These results suggest that host-tumor interactions between host energy balance status and tumor glucose metabolism play an important role in the outcome of early-stage NSCLC. Further study is needed to better understand the underlying mechanism.

## Supporting Information

S1 Table Disease-Free Survival in Multivariable Analyses of Stage I NSCLC Patients (n = 1,197) NSCLC, non-small cell lung cancer; HR, hazard ratio; CI, confidence interval; SUVbsa, maximum standardized uptake value normalized to body surface area
(DOCX)

## References

[1] Vucenik I, Stains JP. Obesity and cancer risk: evidence, mechanisms, and recommendations. Ann N Y Acad Sci. 2012;1271:37–43. 10.1111/j.1749-6632.2012.06750.x 23050962 PMC3476838

[2] Kollarova H, Machova L, Horakova D, Cizek L, Janoutova G, Janout V. Is obesity a preventive factor for lung cancer? Neoplasma. 2008;55(1):71–3. . 18190245

[3] Yang Y, Dong J, Sun K, Zhao L, Zhao F, Wang L, et al Obesity and incidence of lung cancer: a meta-analysis. Int J Cancer. 2013;132(5):1162–9. 10.1002/ijc.27719 . 22777722

[4] Attaran S, McShane J, Whittle I, Poullis M, Shackcloth M. A propensity-matched comparison of survival after lung resection in patients with a high versus low body mass index. Eur J Cardiothorac Surg. 2012;42(4):653–8. 10.1093/ejcts/ezs135 . 22518036

[5] Dahlberg SE, Schiller JH, Bonomi PB, Sandler AB, Brahmer JR, Ramalingam SS, et al Body mass index and its association with clinical outcomes for advanced non-small-cell lung cancer patients enrolled on Eastern Cooperative Oncology Group clinical trials. J Thorac Oncol. 2013;8(9):1121–7. 10.1097/JTO.0b013e31829cf942 23887169 PMC3763835

[6] Leung CC, Lam TH, Yew WW, Chan WM, Law WS, Tam CM. Lower lung cancer mortality in obesity. Int J Epidemiol. 2011;40(1):174–82. 10.1093/ije/dyq134 . 20709687

[7] Yang L, Yang G, Zhou M, Smith M, Ge H, Boreham J, et al Body mass index and mortality from lung cancer in smokers and nonsmokers: a nationally representative prospective study of 220,000 men in China. Int J Cancer. 2009;125(9):2136–43. 10.1002/ijc.24527 . 19585493

[8] Hakimi AA, Furberg H, Zabor EC, Jacobsen A, Schultz N, Ciriello G, et al An epidemiologic and genomic investigation into the obesity paradox in renal cell carcinoma. Journal of the National Cancer Institute. 2013;105(24):1862–70. 10.1093/jnci/djt310 24285872 PMC3866155

[9] Choi Y, Park B, Jeong BC, Seo SI, Jeon SS, Choi HY, et al Body mass index and survival in patients with renal cell carcinoma: a clinical-based cohort and meta-analysis. Int J Cancer. 2013;132(3):625–34. 10.1002/ijc.27639 . 22610826

[10] Dooms C, van Baardwijk A, Verbeken E, van Suylen RJ, Stroobants S, De Ruysscher D, et al Association between ^18^F-fluoro-2-deoxy-D-glucose uptake values and tumor vitality: prognostic value of positron emission tomography in early-stage non-small cell lung cancer. J Thorac Oncol. 2009;4(7):822–8. 10.1097/JTO.0b013e3181a97df7 . 19487964

[11] Vesselle H, Salskov A, Turcotte E, Wiens L, Schmidt R, Jordan CD, et al Relationship between non-small cell lung cancer FDG uptake at PET, tumor histology, and Ki-67 proliferation index. J Thorac Oncol. 2008;3(9):971–8. 10.1097/JTO.0b013e31818307a7 . 18758298

[12] Downey RJ, Akhurst T, Gonen M, Vincent A, Bains MS, Larson S, et al Preoperative F-18 fluorodeoxyglucose-positron emission tomography maximal standardized uptake value predicts survival after lung cancer resection. J Clin Oncol. 2004;22(16):3255–60. 10.1200/JCO.2004.11.109 . 15310769

[13] Paesmans M, Berghmans T, Dusart M, Garcia C, Hossein-Foucher C, Lafitte JJ, et al Primary tumor standardized uptake value measured on fluorodeoxyglucose positron emission tomography is of prognostic value for survival in non-small cell lung cancer: update of a systematic review and meta-analysis by the European Lung Cancer Working Party for the International Association for the Study of Lung Cancer Staging Project. J Thorac Oncol. 2010;5(5):612–9. 10.1097/JTO.0b013e3181d0a4f5 . 20234323

[14] Cavazos DA, deGraffenried MJ, Apte SA, Bowers LW, Whelan KA, deGraffenried LA. Obesity promotes aerobic glycolysis in prostate cancer cells. Nutrition and cancer. 2014;66(7):1179–86. 10.1080/01635581.2014.951738 25264717 PMC4198485

[15] Lynam-Lennon N, Connaughton R, Carr E, Mongan AM, NJ OF, Porter RK, et al Excess visceral adiposity induces alterations in mitochondrial function and energy metabolism in esophageal adenocarcinoma. BMC cancer. 2014;14(1):907 10.1186/1471-2407-14-907 . 25471892 PMC4265417

[16] Consultation WHOE. Appropriate body-mass index for Asian populations and its implications for policy and intervention strategies. Lancet. 2004;363(9403):157–63. 10.1016/S0140-6736(03)15268-3 . 14726171

[17] Lausen B, Schumacher M. Maximally selected rank statistics. Biometrics. 1992:73–85.

[18] Parr CL, Batty GD, Lam TH, Barzi F, Fang X, Ho SC, et al Body-mass index and cancer mortality in the Asia-Pacific Cohort Studies Collaboration: pooled analyses of 424,519 participants. Lancet Oncol. 2010;11(8):741–52. 10.1016/S1470-2045(10)70141-8 20594911 PMC4170782

[19] Calle EE, Rodriguez C, Walker-Thurmond K, Thun MJ. Overweight, obesity, and mortality from cancer in a prospectively studied cohort of U.S. adults. N Engl J Med. 2003;348(17):1625–38. 10.1056/NEJMoa021423 . 12711737

[20] Yoon HH, Lewis MA, Shi Q, Khan M, Cassivi SD, Diasio RB, et al Prognostic impact of body mass index stratified by smoking status in patients with esophageal adenocarcinoma. J Clin Oncol. 2011;29(34):4561–7. 10.1200/JCO.2011.37.1260 21990414 PMC3236656

[21] Yu H, Rohan T. Role of the insulin-like growth factor family in cancer development and progression. Journal of the National Cancer Institute. 2000;92(18):1472–89. . 10995803 10.1093/jnci/92.18.1472

[22] Yu H, Spitz MR, Mistry J, Gu J, Hong WK, Wu X. Plasma levels of insulin-like growth factor-I and lung cancer risk: a case-control analysis. Journal of the National Cancer Institute. 1999;91(2):151–6. . 9923856 10.1093/jnci/91.2.151

[23] Braun S, Bitton-Worms K, LeRoith D. The link between the metabolic syndrome and cancer. Int J Biol Sci. 2011;7(7):1003–15. 21912508 10.7150/ijbs.7.1003 PMC3164150

[24] Hursting SD, Hursting MJ. Growth signals, inflammation, and vascular perturbations: mechanistic links between obesity, metabolic syndrome, and cancer. Arteriosclerosis, thrombosis, and vascular biology. 2012;32(8):1766–70. 10.1161/ATVBAHA.111.241927 . 22815342

[25] Park SY, Lee JG, Kim J, Byun GE, Bae MK, Lee CY, et al Efficacy of platinum-based adjuvant chemotherapy in T2aN0 stage IB non-small cell lung cancer. J Cardiothorac Surg. 2013;8:151 10.1186/1749-8090-8-151 23759129 PMC3684513

[26] Ettinger DS, Akerley W, Bepler G, Blum MG, Chang A, Cheney RT, et al Non-small cell lung cancer. J Natl Compr Canc Netw. 2010;8(7):740–801. . 20679538 10.6004/jnccn.2010.0056

[27] Graham MM, Peterson LM, Hayward RM. Comparison of simplified quantitative analyses of FDG uptake. Nucl Med Biol. 2000;27(7):647–55. . 11091107 10.1016/s0969-8051(00)00143-8

