# Supplementary material for: Influence of Body Mass Index on the Prognostic Value of Tumor ¹⁸F-FDG Uptake in Stage I Non-Small Cell Lung Cancer
Source: PLoS One. 2015 Dec 18;10(12):e0145020. doi: 10.1371/journal.pone.0145020 (PMC4684313; doi:10.1371/journal.pone.0145020)
Supplement: S1 Table — NSCLC, non-small cell lung cancer; HR, hazard ratio; CI, confidence interval; SUVbsa, maximum standardized uptake value normalized to body surface area (DOCX) [file pone.0145020.s001.docx]

***S1 Table.*** *Disease-Free Survival in Multivariable Analyses of Stage I NSCLC Patients (n = 1,197)*

| Variables | Multivariable analysis | | |
| --- | --- | --- | --- |
|  | HR | 95% CI | *P* |
| Age (1-y increase) | 1.02 | 1.00-1.03 | 0.041 |
| Sex, male *vs.* female | 1.51 | 0.90-2.52 | 0.114 |
| Smoking status, |  |  |  |
| Ever-smoker *vs.* never-smoker | 0.82 | 0.51-1.31 | 0.408 |
| Overweight/obesity *vs.* normal weight | 0.62 | 0.45-0.86 | 0.004 |
| Histologic cell type |  |  |  |
| Squamous *vs.* non-squamous | 0.85 | 0.56-1.27 | 0.430 |
| Tumor differentiation |  |  |  |
| Poor *vs.* well or moderate | 1.28 | 0.82-1.96 | 0.272 |
| Pathological tumor size (cm) | 1.09 | 0.90-1.31 | 0.355 |
| Lymphovascular invasion | 2.42 | 1.73-3.36 | < 0.001 |
| Visceral pleural invasion | 2.29 | 1.57-3.33 | < 0.001 |
| Limited resection less than lobectomy | 1.29 | 0.63-2.65 | 0.476 |
| Tumor SUVbsa (continuous, log_2_ scale) | 1.69 | 1.40-2.04 | < 0.001 |

NSCLC, non-small cell lung cancer; HR, hazard ratio; CI, confidence interval; SUVbsa, maximum standardized uptake value normalized to body surface area
